# Parallelized Ultrasound-Guiding for Enhanced Light Delivery within Scattering Media

**DOI:** 10.1021/acsphotonics.4c01398

**Published:** 2024-11-15

**Authors:** Blanca Mestre-Torà, Martí Duocastella

**Affiliations:** † Department of Applied Physics, University of Barcelona, 08028 Barcelona, Spain; ‡ Institut de Nanociència i Nanotecnologia (In2UB), University of Barcelona, 08028 Barcelona, Spain

**Keywords:** acousto-optics, remote light control, deep light focusing, scattering, inertia-free light control

## Abstract

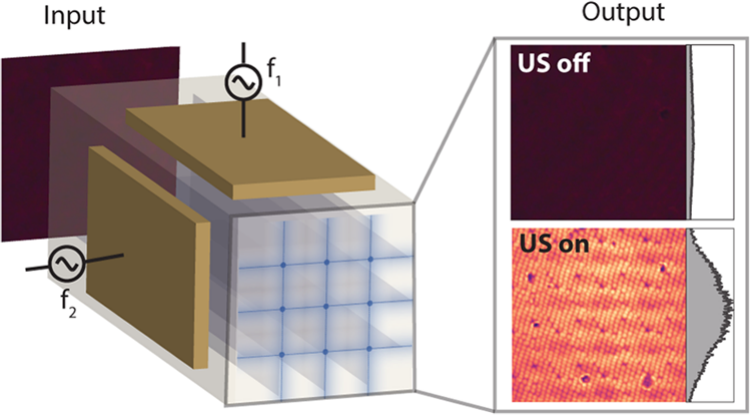

The delivery of light over an extended area within a sample forms the basis of biomedical applications that are as relevant as photoacoustic tomography, fluorescence imaging, and phototherapy techniques. However, light scattering limits the ability of these methods to reach deep regions within biological tissues. As a result, their operational range remains confined to superficial areas of samples, posing a significant barrier to effective optical treatment and diagnosis. Here, we propose an approach to address this issue and enhance light delivery across an extended region inside scattering samples. Our strategy involves using ultrasound to directly modulate the optical properties of the sample, generating refractive index gradients that act as embedded optical waveguides. By employing two perpendicularly oriented piezoelectric plates, several parallel waveguides can be simultaneously formed within the sample, allowing light to be guided over a wide area (3 × 3 mm^2^ in current experiments). Supported by Monte Carlo simulations, we demonstrate that ultrasound-light-guiding can enhance the intensity of light delivered inside scattering samples with an optical thickness of 2.5 and 12.5 by up to a factor of 700 and 42%, respectively. As a proof-of-concept, we demonstrated the ability of our approach to irradiate nanoparticles located within a scattering sample at light intensities that are not possible without ultrasound.

## 1 Introduction

Light-based methods based on light delivery across a broad region are of prime importance in biomedical and life sciences.^1−3^ Examples include photoacoustic tomography^4,5^ and phototherapy techniques,^6,7^ which can operate at depths of some millimeters inside biological tissues.^8^ While this depth is superior to that of techniques based on focused light, such as confocal or two-photon microscopy, it remains insufficient for comprehensive diagnosis and localized treatments. Deeper light delivery, which is key for extending the operational range of these techniques beyond the outer layers of a sample, is limited by scattering.^9,10^ Upon entering a scattering medium, light intensity typically decreases exponentially with a parameter known as optical thickness (τ), a regime referred to as ballistic transport.^11^ This parameter depends on the scattering properties of the medium and the depth and indicates the number of scattering events the light undergoes. For most biological tissues, the amount of light delivered beyond τ > 10, when reaching a diffusive regime,^12^ is too low for effective imaging or treatment.

Efforts to improve light delivery over wide regions within scattering samples include using miniaturized endoscopes.^13,14^ These fiber-bundle devices offer an unlimited penetration depth but at the cost of increased invasiveness. Adjusting the wavelength and increasing the power of the light source have also proved efficient in enhancing the light delivery depth. However, these strategies have a fundamental limit. Even at the near-infrared wavelengths where scattering is the least, the amount of power required to go further deep inside a sample can be too high for practical implementations. Additionally, the spreading of light at nontargeted regions can induce phototoxic effects.^15,16^ Other methods to compensate for light scattering include optical wavefront shaping techniques, which are based on reversing the deterministic scattering process.^17,18^ However, these techniques require a full characterization of the scattering medium, which can be a process that is too slow for living systems or other rapid processes. Alternatively, it is possible to use ultrasound waves to encode or tag photons, a method known as acousto-optic tomography.^19^ Unfortunately, the low efficiency of photon encoding typically challenges the detection of tagged photons over background noise.^20,21^ A newly developed approach that offsets these trade-offs consists of using ultrasound to induce a refractive index modulation directly inside the sample of interest, which effectively acts as an embedded lens or waveguide that bends photons toward regions with a high refractive index. As a result, scattered and ballistic photons can be redirected and guided to deep sections—deeper than traditional external focusing elements.^22−24^ So far, the main implementation of ultrasound-waveguiding involves using piezoelectric resonant cavities to focus light into a single spot.^24−27^ Still, the suitability of ultrasound-waveguiding to enhance light delivery over an extended area has not yet been explored.

In this work, we fill this void and maximize the ultrasound-light-guiding phenomenon over millimeter-squared areas within the scattering media. Our method is based on parallelizing the generation of ultrasound waveguides by using two perpendicular piezoelectric plates, each emitting a traveling plane wave orthogonal to the light propagation direction. When they interfere, a grid of two-dimensional (2D) sinusoidal refractive index distributions is formed.^28,29^ Such a distribution acts as a grid of parallelized waveguides, whose number depends on the piezoelectric plate driving frequency and its extent. As our results and simulations demonstrate, such parallelization allows for up to a 700 and 42% enhancement of the light intensity delivered over an area of 3 × 3 mm^2^ at a depth of 2 cm within a 2.9 and 12.5 mean free path (MFP) scattering sample, respectively.

## 2 Parallelized Ultrasound-Waveguiding

**Figure 1:**
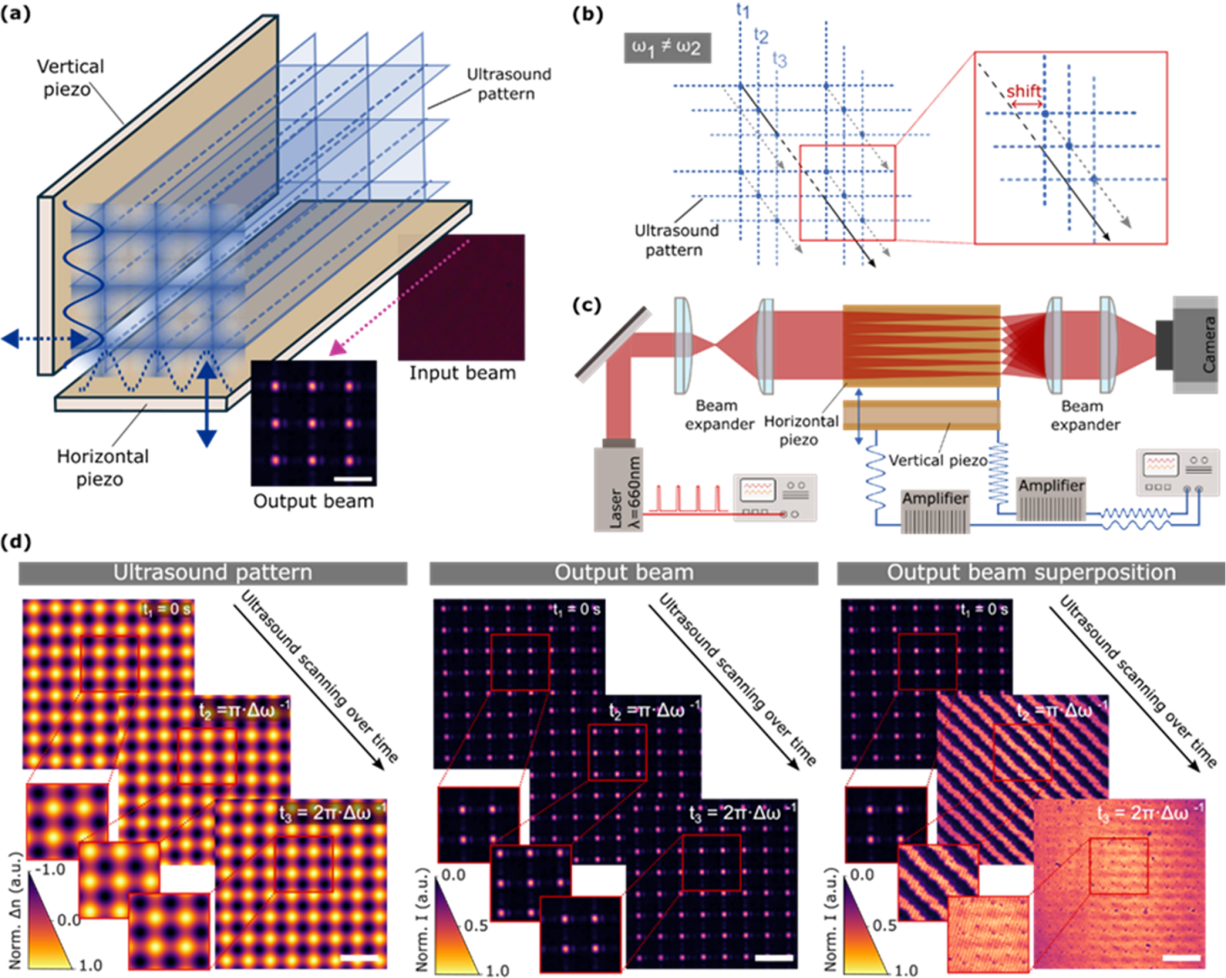
Formation of multiple parallel ultrasound-generated waveguides. (a) Sketch of the configuration of the piezoelectric plates for the generation of ultrasound-generated waveguides. The scale bar is 150 μm. (b) Scheme of the scanning of the ultrasound pattern when both piezoelectric plates oscillate at a slightly different frequency. (c) Scheme of the experimental setup employed in this work. A pulsed laser is directed toward the perpendicularly placed piezoelectric plates, where the beam is focused down to multiple spots. (d) Images of the ultrasound (left) and light scanning (middle) at different time instances and superposition (maximum intensity projection) of the scanning light beam at different times (right). Scale bars are 300 μm.

The gist of our approach for deep light delivery across a wide area consists of generating a tailored ultrasound field inside a sample that acts as multiple embedded optical waveguides. To this end, we used two orthogonal piezoelectric plates, as shown in Figure 1a. When vibrating at one of the resonant frequencies of the piezoelectric plate (see the Supporting Information and Figure S1), each of them generates a traveling ultrasound plane wave,^30^ given by
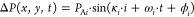
1where the subindex *i* indicates the propagation direction (*x* and *y*), *P*_A_ is the amplitude of the pressure wave, and κ, ω, and ϕ are the wavenumber, angular frequency, and phase, respectively. Such a change in pressure leads to local variations in the density of a medium and, consequently, its refractive index.^31−33^ As shown in Figure S2, for the pressure values generated by the ultrasound wave, such a relationship is linear. Thus, upon the interference of the two orthogonal waves, a grid of sinusoidal gradient refractive index (GRIN) waveguides is formed within the medium, which can be written as
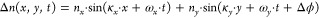
2where *n*_*i*_ is the change in the refractive index induced by ultrasound, and Δϕ is the phase difference between the two waves. Notably, when a pulsed Gaussian light beam of duration *T*  is transmitted through the modulated medium and provided *T* ≪ 2π/ω_*i*_, the light is preferentially directed toward regions of higher instantaneous refractive index. Due to the specific geometry of the ultrasound waves, these regions form an array of GRIN waveguides.^34^ As a result, at the output of the interaction region, light focuses into multiple spots, one for each waveguide, as shown in Figure 1a. By adjusting the delay time between laser pulses and ultrasound waves, it is possible to change the spatial location of the light spot array. This unique feature allows for dynamic sample scanning; see Figure 1b. Specifically, by driving the piezoelectric plates with slightly different frequencies, their interference creates an ultrasound field that is periodically moving. The corresponding period *T*_beating_ depends on the difference in frequencies, a phenomenon known as frequency beating,^35^ and can be written as
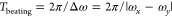
3

In the current experiments, we used a frequency difference of the order of kHz, resulting in *T*_beating_ below 1 millisecond. Interestingly, it is also possible to illuminate the ultrasound-modulated sample with continuous light. In this case, an average effect of the grid of sinusoidal waveguides is observed. Typically, this results in a slight deterioration of contrast relative to pulsed illumination (see the Supporting Information and Figure S3).

To implement the parallelized ultrasound-waveguiding system in practice, we used the setup shown in Figure 1c (see Section 5 for further details). It features two orthogonal piezoelectric plates, each driven at a frequency of around 8.8 MHz and which are placed inside a transparent chamber filled with the medium of interest—water/milk dilutions in experiments herein—a 660 nm pulsed laser diode, and a camera for visualization. The use of water/milk mixtures offers the possibility to easily select the scattering properties of the medium (see Section 5, Supporting Information, and Figure S4).

## 3 Results

### 3.1 Light-Guiding in Nonscattering Media

Figure 1d shows an example of the light-guiding effects within a nonscattering medium using pulsed illumination at three different time instances. As described in eq 3, such a time determines the instantaneous ultrasound pattern that interacts with the light pulses. The result of this interaction is the formation of multiple beam spots at the output of the medium. As expected, for each time instance, a spatial shift of the beamlets is experimentally observed. By accumulating these multispot distributions over time, the light delivered at the medium output progressively accumulates. Importantly, after 0.1 ms, the superposition of light, calculated as the maximum intensity projection, leads to an intensity enhancement of a factor of 950% compared to nonultrasonically guided light. Note that this type of maximum intensity effect naturally occurs in threshold-driven processes, such as photodynamic therapy (PDT) and other light–matter interactions. Therefore, in our experiments using a nonscattering medium, parallelized ultrasound-waveguiding results in a factor of 10 enhancement in light delivery.

**Figure 2:**
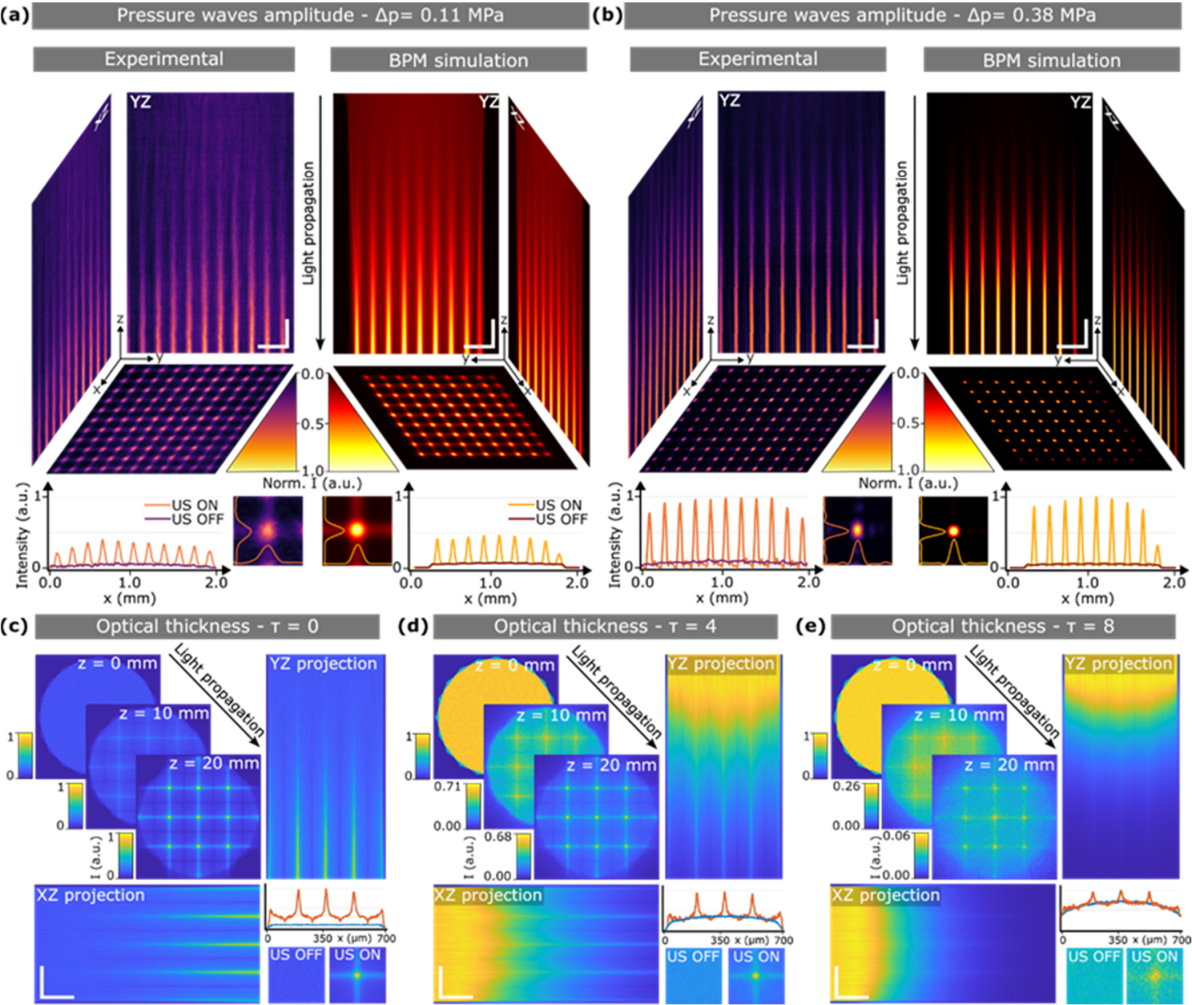
Experimental measurements and simulations of the parallelized light-guiding with ultrasound waves. Experiment and BPM simulations of light propagation inside ultrasound-modulated homogeneous media at an amplitude pressure of 0.11 (a) and 0.38 MPa (b). The plots show the corresponding intensity profile normalized with respect to panel (b). The insets show the simulated and experimental intensity images at the intersection point between two ultrasound maxima. Horizontal scale bars are 300 μm, and vertical scale bars are 2 mm. Intensity images correspond to three-dimensional (3D) Monte Carlo simulations of the beam propagation inside the ultrasound-modulated media at optical thicknesses of 0 (c), 4 (d), and 8 (e). For each case, the beam at propagation distances of *z* = 0 mm, *z* = 10 mm, and *z* = 20 mm is shown. *YZ* and *XZ* propagation projections with the normalized output beam intensity profile (orange data correspond to ultrasound ON, blue data correspond to ultrasound OFF). The insets show zoomed-in intensity images at the intersecting point between two ultrasound maxima. Horizontal scale bars are 3 mm, and vertical scale bars are 200 μm.

A more in-depth analysis of the ultrasound-enabled light-guiding phenomenon is shown in Figure 2. In this case, we measured the beam propagation inside a homogeneous medium modulated with ultrasound at two different pressure amplitudes: 0.11 and 0.38 MPa (see the Supporting Information and Figure S5). Notably, as light propagates within the medium, it is progressively bent and guided toward multiple spots. This effect is more pronounced as the amplitude pressure increases. Indeed, for 0.38 MPa, light intensity confinement after the output of the modulated region (2 cm) is a factor of 2 higher compared to 0.11 MPa. Interestingly, higher-pressure values result in higher light confinement after shorter light propagation, as shown in Figure S6. These results are in excellent agreement with simulations using the beam propagation method (BPM),^36^ as shown in Figures 2 and S6. The only input parameters were the refractive index gradient of the medium—calculated with eq S1 using experimental measurements of the pressure with a needle hydrophone—and the wavelength and size of the incident Gaussian beam (see Section 5). This confirms that our ultrasound field acts as an array of gradient refractive index waveguides, helping to deliver light over an extended area.

### 3.2 Light Delivery Enhancement in Scattering Media

The key question that remains is how advantageous it is to use ultrasound for guiding light deep into the scattering samples. To this end, we first used three-dimensional (3D) Monte Carlo simulations based on the photon packet method^37^ (see Section 5). This is the standard method to simulate light transport in scattering media. As expected, for light propagation in an ultrasound-modulated medium with a pressure of 0.38 MPa and negligible scattering, the results are identical to those calculated with the BPM method (Figure 2b,2c). Thus, as light propagates within the homogeneous medium, it is progressively focused down to multiple spots. The same trend occurs when simulating light propagation within scattering media (Figure 2d,2e). Despite the light attenuation induced by scattering, a notable quantity of photons can still reach the output of the medium, guided by ultrasound. The confined multispots are still visible, even after an average of 8 scattering events (optical thickness τ = 8). More importantly, the peak light intensity delivered at the output of the medium is increased by a 2.5 and 1.33 factor for τ = 4 and τ = 8, respectively, compared to the absence of ultrasound (see Supporting Information and Figure S7 for further details of light intensity enhancement using ultrasound-guiding). Such light enhancement is also consistent with results obtained using a modified BPM method that accounts for scattering (Figure S8).

**Figure 3:**
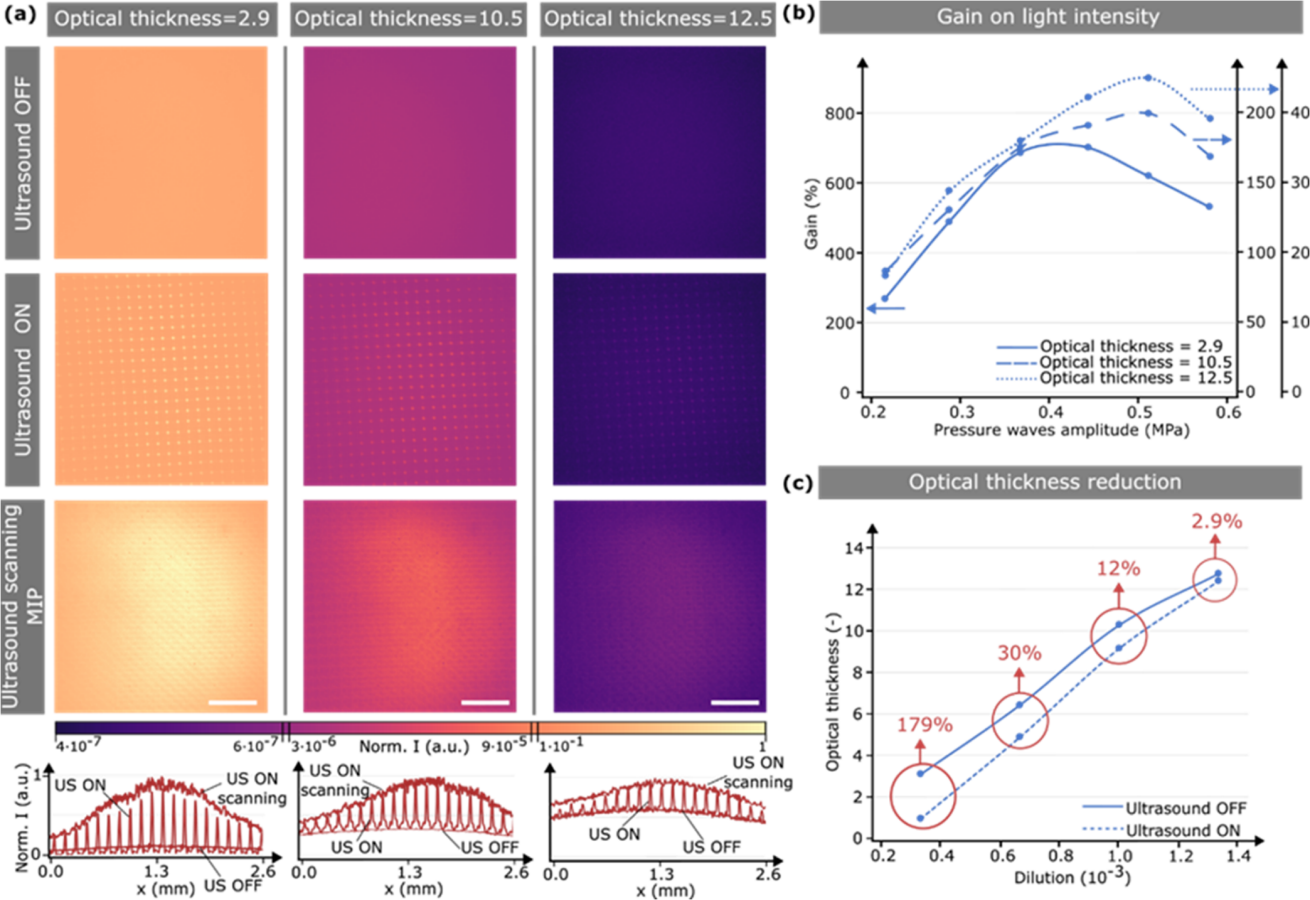
Light intensity enhancement in scattering media. (a) Images of a laser beam after passing through scattering media with optical thicknesses of τ = 2.9 (left), τ = 10.5 (middle), and τ = 12.5 (right) when the ultrasound is OFF (top), ON (middle), and ON superposition (maximum intensity projection) of a scanning sequence (bottom). The ultrasound pressure amplitude was 0.5 MPa. The bottom plots show the corresponding beam intensity profile at its vertical center for each condition. Scale bars are 700 μm. (b) Plot of gain on the light intensity at the output of different scattering media as a function of the pressure wave amplitude. Lines are guides for the eye. (c) Plot of the optical thickness of different water/milk dilutions (v/v) when the ultrasound is OFF and ON—scanning mode. Lines are guides to the eye. The red arrows indicate the reduction of the optical thickness of the medium when ultrasound is activated.

An experimental evaluation of light-guiding enhancement by ultrasound is shown in Figure 3a. In this case, we analyzed three different scenarios, corresponding to ultrasound OFF and ultrasound ON with and without scanning, respectively. For each case, we captured images of the intensity of a pulsed laser beam after passing through media with different optical thicknesses, ranging from 2.9 to 12.5. As expected, when the ultrasound is OFF, the beam intensity at the output of the scattering medium is reduced relative to the initial intensity. This effect increases with optical thickness. Notably, the use of ultrasound helps to partially compensate for such light attenuation. As shown in Figure 3a, middle row, ultrasound-induced guiding results in an array of spots. For all of the optical thicknesses analyzed, the intensity of each spot is significantly higher than that of the surrounding area and, more importantly, than the intensity without ultrasound (see inset profiles). To extend this enhancement across a broader area, we captured a sequence of images using the dynamic scanning strategy described in Section 2. For each optical thickness, the superposition of these images is shown in Figure 3a, bottom row. Compared to ultrasound OFF, there is a significant enhancement in the light delivered over an area of 3 × 3 mm^2^. The extent of this effect is reduced with the optical thickness. In other words, the benefits of ultrasound are less pronounced for samples exhibiting a higher scattering. Still, ultrasound-enabled light delivery at an optical thickness of 12.5 remains clearly superior to that when the ultrasound is off.

Figure 3b shows the quantification of the gain in light delivery offered by ultrasound at different optical thicknesses and amplitude pressures. We took all measurements at the output of the piezoelectric plates and defined gain as the difference in light intensity at that position with (*I*_ON_) and without (*I*_OFF_) ultrasound, relative to the latter: gain = (*I*_ON_ – *I*_OFF_)/*I*_OFF_. For the three optical thicknesses analyzed, the trend is similar: increasing the pressure amplitude of the ultrasound waves initially leads to a significant enhancement in light intensity, peaking at 0.4–0.5 MPa, after which the gain slightly decreases. This behavior is due to the focusing nature of ultrasound-light-guiding. As the amplitude pressure increases, stronger light focusing occurs, up to the point where focusing takes place right at the measurement plane (maximum gain). From this value, further increasing the pressure leads to focusing within the medium, that is, before reaching the measurement position. Thus, the collected light is progressively defocused and less intense (see the Supporting Information and Figure S6). Note that, had the light continued to propagate in an ultrasound-modulated medium, further focusing would have occurred, following a focusing/defocusing sequence similar to that observed in GRIN waveguides. Remarkably, a 700% gain in light intensity can be reached for an optical thickness of 2.9. As previously observed, the gain decreases with scattering, but still impressive gain factors of 200 and 42% can be obtained at an optical thickness of 10.5 and 12.5, respectively.

So far, we have analyzed ultrasound-induced enhancement in light delivery at a certain depth. A different, and arguably more realistic, estimation of the benefits of ultrasound involves computing the induced reduction in optical thickness τ. Note that τ = μ_S_*d*, where μ_S_ and *d* are the scattering coefficient and thickness of the medium, respectively. Thus, quantifying the reduction of τ provides a direct measurement of the effective gain in transparency (decrease in μ_S_) or, equivalently, gain in penetration depth (decrease in the medium thickness). Figure 3c shows the measured optical thickness for different milk/water dilutions with ultrasound OFF and ON. In agreement with previous results, the reduction in optical thickness is more significant for lower scattering media. It drops from 2.9 to 1.7 for a dilution of 3 × 10^–4^ (v/v) when using ultrasound. In other words, under these conditions, light could be delivered at a depth 179% deeper than that without ultrasound. Similarly, for dilutions with an optical thickness of 10.2 and 12.5, ultrasound renders the media 12 and 2.9% more transparent, respectively. Note that, despite the impressive gain in intensity previously reported (Figure 3b), the increase in penetration depth is more limited. This is expected given the exponential relationship between intensity and optical thickness, highlighting the inherent challenge of increasing light penetration within scattering media. Still, even a reduction of optical thickness by just a few percentage points can significantly extend the effectiveness of treatments such as photodynamic or photothermal therapies,^6,7^ as well as the characterization range of diagnostic tools like photoacoustics.^4,5^

### 3.3 Proof-of-Concept

**Figure 4:**
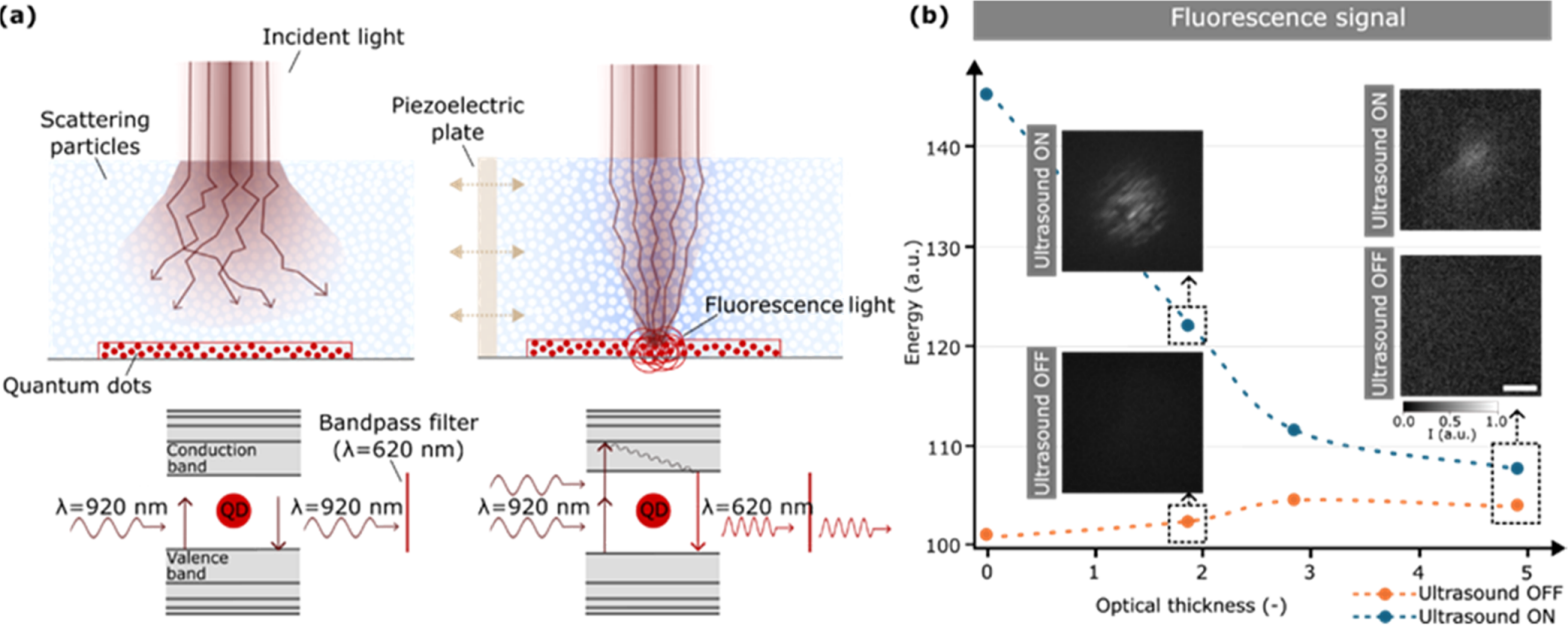
Two-photon ultrasound-induced excitation of quantum dots immersed in scattering media. (a) Schematic of the light propagation in a scattering medium without (left) and with (right) ultrasound and its subsequent excitation of fluorescent red QDs. (b) Plot and images of the emitted fluorescence signal generated by red QDs immersed in scattering media at various optical thicknesses when ultrasound is OFF and ON—scanning mode. Note that both sets of images in panel (b) were normalized. Dashed lines are guides for the eye. Scale bar: 500 μm.

As a proof-of-concept of the potential of ultrasound-guided light, we conducted an experiment that mimicked the conditions of photodynamic therapy (PDT). PDT is based on the broad-area irradiation of nanoparticles (photosensitizers) located within biological tissue upon which a chemical reaction occurs. In our experiment, we used a layer of fluorescent red quantum dots (CdSe/ZnS core–shell, emission wavelength λ = 620 nm) as our nanoparticles,^38,39^ which were immersed in a complex medium (see Section 5). As an incident light source, we used an ultrashort infrared laser (λ = 920 nm, 100 fs pulse width) directed toward the scattering sample (see Section 5). Importantly, the red quantum dots emit red fluorescent light only when excited with a wavelength between 400 and 550 nm. Therefore, only focused ultrashort pulses at 920 nm possess the intensity needed to induce a two-photon absorption process and generate red fluorescence (Figure 4a). These conditions are similar to those of PDT, where light intensity above a certain threshold is required to induce drug activation. As shown in Figure 4b, the absence of ultrasound results in no emitted fluorescence. In other words, without ultrasound, there is not enough light intensity at the plane where the quantum dots are located to induce two-photon absorption. This situation is completely reversed with ultrasound ON. In this case, the increase in light delivery results in fluorescence emission, even within scattering samples with optical thicknesses of τ = 1.9 and τ = 5.0. As previously observed, the overall energy delivered at the sample decreases with scattering and so does the emitted fluorescence. In this particular case, given the nonlinear interaction between nanoparticles and light, this effect is more pronounced. These results prove that ultrasound is a feasible method for enhancing the photoexcitation of particles immersed within scattering constructs.

## 4 Conclusions

Ultrasound waves can be used to guide and enhance light delivery inside scattering media over an extended area. Our method is based on combining the acousto-optic effect with the interference of ultrasound waves propagating in orthogonal directions. The result is the effective formation of an array of optical waveguides embedded within a sample. By proper synchronization with pulsed illumination, such an array enables uniform and extended light delivery over a wide area—up to 3 × 3 mm^2^—within scattering media. Supported by Monte Carlo simulations, our experimental results show that ultrasound-waveguiding can enhance light delivery by up to 700 and 200% within scattering samples with optical thicknesses of 2.9 and 10.5, respectively. This enables enhancing the delivering light to nanoparticles embedded in scattering media, which is paramount for diagnosis and therapeutic techniques.

The maximum amplitude of the pressure waves employed is 0.5 MPa (see the Supporting Information and Figure S2). Higher-pressure values would help to act as more efficient waveguides, but high-power ultrasound can be damaging to biological samples. Still, the damage threshold defined by the Food and Drug Administration (FDA) is 5.4 MPa rarefaction pressures.^40^ Therefore, ultrasound-light-guiding at the pressures reported herein can be considered a noninvasive method. The current implementation of ultrasound-waveguiding features two orthogonal piezoelectric plates of small size and closely located. In this configuration, sample placement can be cumbersome. However, this issue could be alleviated by increasing the separation between plates and increasing the driving radio frequency power, necessary to compensate for the attenuation of ultrasound over distance. Importantly, the overall system is easy to implement and has low cost, with the driving electronics being the most expensive components.

Overall, our results highlight the advantage of using ultrasound to enhance light delivery over wide areas deep in highly scattering samples. We believe that integrating this approach with state-of-the-art light-based methods such as PAT, PDT, and photoacoustic microscopy can open the door to extending the operational depth of these techniques without sacrificing their core advantages.

## 5 Materials and Methods

### 5.1. Experimental Setup

The main components of the setup are depicted in Figure 1c. A diode laser with a wavelength of 660 nm (Coherent Obis) was used as the light source. The laser intensity amplitude is modulated at a frequency of 8.8 MHz, effectively achieving laser pulses with a duration of around 10 ns. The laser beam was expanded with a 4f system of 3.6× magnification and then directed toward the transparent chamber containing the perpendicularly placed piezoelectric plates, 2 cm long and 1 cm wide, made of the piezoelectric material PZT (lead zirconate titanate). The multiple-focused light beam is then directed toward a charged-coupled device (CCD) camera (Thorlabs, DCU224M) via a microscope with 1.5× magnification. To generate the ultrasound traveling waves in the medium, the piezoelectric plates were continuously driven at a frequency of around 8.8 MHz using an arbitrary waveform generator (AWG) (Siglent, SDG6022X) and a high-power amplifier (Minicircuits, LZY-22+), leading to pressure waves with amplitudes of up to 0.5 MPa. The synchronization signal of the AWG was used to trigger the laser pulses.

### 5.2. Scattering Sample Preparation and Characterization

The scattering phantoms used in this work were prepared by diluting fat milk (scattering coefficient of μ_S_ = 30 mm^–1^^41^ and anisotropy factor of *g* = 0.94^41^) in pure water. The water/milk mixture enables the easy tuning of the scattering properties by simply changing the concentration of milk in the solution. The highest dilution of milk was obtained at a concentration of 14:1000. The scattering properties of the prepared samples were determined by analysis of the light attenuation as a function of thickness, from which the scattering coefficient and optical thickness of the mixture are obtained (see the Supporting Information and Figure S4).^42^

### 5.3. Beam Propagation Method (BPM) Simulations

The propagation of light inside a homogeneous medium was simulated using the beam propagation method implemented in Python: Light Pipes for Python.^36^ This algorithm is used to estimate the propagation of a light beam in a medium where diffraction is crucial. The simulation parameters for the beam propagation in an ultrasound-modulated homogeneous medium are 660 nm wavelength, incident Gaussian beam with a 1500 μm waist, a propagation distance of 2 cm, a refractive index amplitude distribution described by eq 1—with an amplitude ranging from *n*_A_ = 2 × 10^–5^ to *n*_A_ = 8 × 10^–5^, the values were calculated with eq S1 using the experimental measurements of the pressure with a needle hydrophone, a frequency of 8.8 MHz, and a static refractive index of 1.33 (water).

### 5.4. 3D Monte Carlo Simulations

The propagation of light inside a scattering medium was simulated using a recently published Monte Carlo implementation.^37^ The algorithm is based on the photon packet method, where individual photons are emitted from a defined light source and propagated through a medium. As the photons travel through the media, they experience scattering and absorption interactions, with a probability given by the scattering coefficient (μ_S_) and the absorption coefficient (μ_a_). When a scattering event occurs, the photon propagation direction is randomly altered within an angular range determined by the scattering anisotropy factor (*g*). By simulation of the propagation of millions of photons, the algorithm provides detailed insights into light propagation within the medium, including its spatial distribution, penetration depth, and fluence. The Monte Carlo implementation used requires a local specification of the medium properties—scattering coefficient, absorption coefficient, scattering anisotropy factor, and refractive index (*n*)—on a tetrahedron grid geometry. For simulations of light propagation within nonmodulated media, the distribution of the refractive index is set to be constant across all voxels in the mesh grid. Instead, simulations with ultrasound-modulated media make use of the refractive index distribution corresponding to eq 2. In this work, different scattering coefficients ranging from 0.5 to 5 cm^–1^ were used. In all cases, absorption was considered negligible^41^ and the anisotropy factor value selected was *g* = 0.94.^41^ The photon source had a length of 680 μm and a small divergence of σ = 6 × 10^–3^ mm. For ultrasound focusing, the refractive index distribution reported in eq 1 was used with *n*_A_ = 1 × 10^–5^ at a frequency of 8.8 MHz. For the simulations of light propagation within nonmodulated media, the distribution of the refractive index was set to be homogeneous, that is, *n*_A_ = 0. The static refractive index of water was considered. In this case, the simulated region was 2 cm long and 700 μm wide, with a grid size of 4 μm. The Monte Carlo algorithm was implemented in Matlab.

### 5.5. Quantum Dot Sample Preparation

The samples containing the quantum dots were prepared using agar–agar powder (Sigma-Aldrich) diluted in deionized water with a 1:1000 (w/v) ratio. The agar–agar was diluted by boiling the mixture with a hot plate and sonicating it with a magnetic stir bar. Then, the quantum dots were mixed with the agar–agar dilution in a 1:2 (v/v) ratio, piped in a glass slide, and covered with a coverslip.

### 5.6. Fluorescence Emission Setup

The setup used for the main experiments was adapted by adding or replacing some components. The laser source is a femtosecond laser with a wavelength of 920 nm (Coherent, AXON 920). The laser beam is reduced using a 4f system with a 5× magnification. In the detection branch, a beamsplitter (cutoff wavelength λ = 805 nm) and a bandpass filter (hard-coated bandpass filter, λ = 620 nm) were incorporated to filter out the excitation light and collect the fluorescence emission.

## Supporting Information Available

Piezoelectric plate characterization (Section S1); refractive index modulation and pressure wave amplitude relationship (Section S2); intensity enhancement with continuous light (Section S3); experimental measurement of the scattering coefficient (Section S4); beam propagation measurement (Section S5); beam guiding at different pressure wave amplitudes (Section S6); light intensity enhancement with ultrasound-waveguiding (Section S7); and beam propagation method (BPM) simulations in scattering media (Section S8) (PDF)
