# Supplementary material for: Parallelized Ultrasound-Guiding for Enhanced Light Delivery within Scattering Media
Source: ACS Photonics. 2024 Nov 15;11(12):5161–9. doi: 10.1021/acsphotonics.4c01398 (PMC11660215; doi:10.1021/acsphotonics.4c01398)
Supplement: Supplementary file 1 — ph4c01398_si_001.pdf [file ph4c01398_si_001.pdf]

Supplementary Information:

Parallelized ultrasound-guiding for enhanced light delivery within scattering media

Blanca Mestre-Torà[a], Martí Duocastella[a,b,*]

[a] Department of Applied Physics, University of Barcelona, 08028 Barcelona, Spain
[b]Institut de Nanociència i Nanotecnologia (In2UB), University of Barcelona, 08028 Barcelona, Spain
* Corresponding author e-mail: marti.duocastella@ub.edu

Supplementary Notes

S1 - Piezoelectric plates characterization 2

S2- Refractive index modulation and pressure wave amplitude relationship........ 3

S3- Intensity enhancement with continuous light 4

S4- Experimental measurement of the scattering coefficient........ 5

S5- Beam propagation measurement 6

S6- Beam guiding at different pressure wave amplitudes........ 7

S7- Light intensity enhancement with ultrasound waveguiding 8

S8- Beam Propagation Method (BPM) simulations in scattering media 10

Supplementary Figures

Figure S1. Calibration of the piezoelectric plates. 2

Figure S2. Relationship between the refractive index variation and pressure changes in water. 3

Figure S3. Beam visualization at the medium output with pulsed and continuous illumination . 4

Figure S4. Experimental measurement of the scattering coefficient of the water/milk mixtures.5

Figure S5. Schematic of the set-up used to measure the beam propagation... 6

Figure S6. Light guiding in ultrasound-modulated media at different pressure amplitudes........ 7

Figure S7.1. Monte Carlo simulation in a scattering medium of τ = 4........ 8

Figure S7.2. Monte Carlo simulation in a scattering medium of τ = 8........ 9

Figure S8. BPM simulations in scattering media with ultrasound waveguiding system........ 10

References........ 11

S1 - Piezoelectric plates characterization

The amplitude pressure of the ultrasound generated by the piezoelectric plates determines the refractive index gradient induced within the sample, and consequently, the effectiveness of the induced light-guiding effect. To characterize the piezoelectric plate response and the optimal conditions for operation, we experimentally measured the amplitude of ultrasound waves generated in water at various frequencies and input voltages. We conducted these measurements by using a needle hydrophone placed 1 cm away from the piezoelectric plate. As shown in Figure S1a, the plate efficiently emitted at specific resonant frequencies, with the fundamental frequency located at 1.57 MHz. Furthermore, for each resonant frequency, increasing the amplitude of the driving input voltage resulted in a linear increase in the amplitude of the ultrasound waves, as shown in Figure S1b.

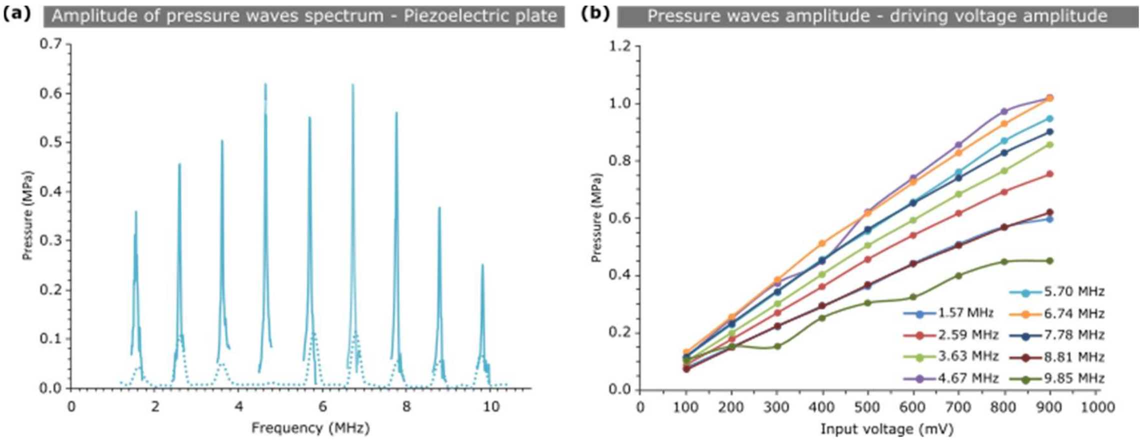

***Figure S1*.** **Calibration of the piezoelectric plates**. (a) Plot of the spectrum of the pressure wave amplitude generated by one of the ultrasound transducers across a range of frequencies from 1 MHz to 10MHz. (b) Plot of the dependence of the pressure wave amplitude on the input driving voltage before amplification for each of the piezoelectric resonant frequencies.

S2- Refractive index modulation and pressure wave amplitude relationship

The local variation of pressure in water produces a local variation of the refractive index. The relationship between these parameters can be explicitly written as [1]:

$$n(\tilde{p}) = \sqrt{\frac{1+2(a_9+(a_9+a_{10})\tilde{p}+(a_7+a_{10})\tilde{p}^2+a_7\tilde{p}^3)}{1-(a_9+(a_9+a_{10})\tilde{p}+(a_7+a_{10})\tilde{p}^2+a_7\tilde{p}^3)}} \quad \text{(S.1)}$$

where n corresponds to the refractive index, a_i are the dimensionless coefficients given in IAPWS [2], and $\tilde{p}$ is defined as a dimensionless pressure produced by acoustic fluctuations. Thus, an estimation of the pressure variation in water can be obtained by knowing the induced changes in refractive index, as shown in Figure S2. Considering a pressure wave amplitude of $\Delta p = 0.3\ MPa$, it yields a variation in the refractive index of $\Delta n = 4 \cdot 10^{-5}$ in the medium (see Results section in the main text).

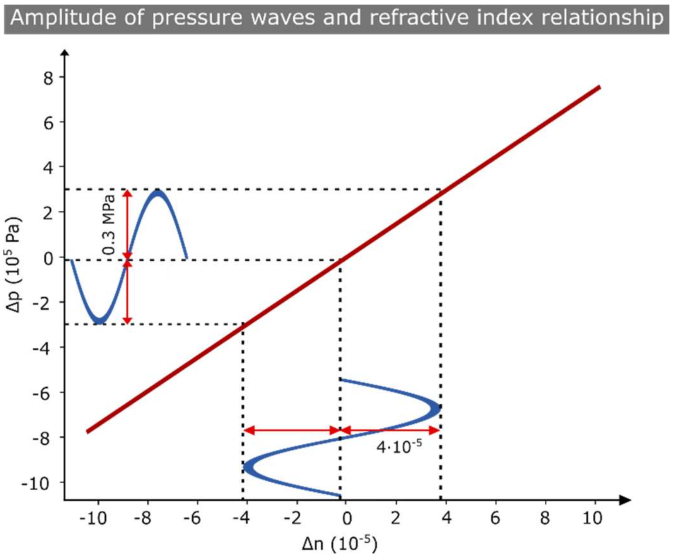

***Figure* S2. Relationship between the refractive index variation and pressure changes in water.** Plot of the variations in pressure versus the change in refractive index. At the conditions considered herein, there is a linear relationship between the two.

S3- Intensity enhancement with continuous light

All ultrasound waveguiding experiments reported in the main text employed pulsed light. However, it is also possible to use continuous light. In this case, ultrasound still produces a gain in intensity, as shown in Figure S3-right. Given that the ultrasound waves are traveling, the multi-spot pattern is continuously moving. By using continuous light, the intensity average of these patterns is obtained. This can be readily captured with a camera, which typically features an exposure time much longer than the ultrasound propagation time. In fact, to avoid averaging effects, as in Figure S3-left, very short pulses are needed (about 10 ns). There is a slight deterioration of contrast when using continuous light compared to pulsed light. Still, the regions where light is focused – diagonal stripes when both piezoelectric plates are driven at the same frequency – are clearly visible (Figure S3-left).

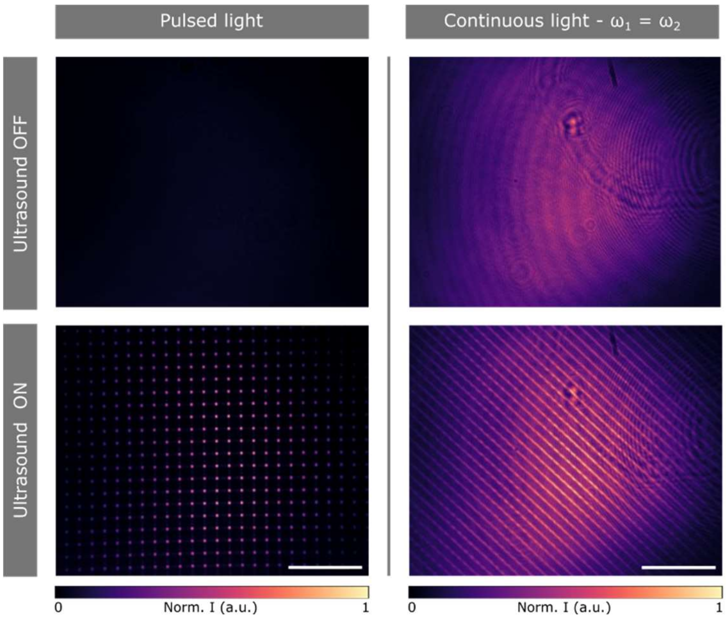

Figure S3. **Ultrasound guiding with pulsed and continuous illumination**. Optical images of a laser beam at the output of the ultrasound-modulated medium with pulsed (left) and continuous (right) light when driving both orthogonal piezoelectrics with the same frequency of 8.8 MHz. The first row of images represents the ultrasound OFF scenario, and the second row shows the ultrasound ON case. Scale bar 1 mm.

S4- Experimental measurement of the scattering coefficient

To determine the scattering coefficient (μ_s) of the water/milk mixtures, we measured the light attenuation as a function of the medium thickness. Specifically, we placed a mirror inside a reservoir filled with the turbid medium and collected the reflected light intensity at different mirror-reservoir distances, as shown in Figure S4. Considering the single-scattering regime, the observed light attenuation as light travels a longer distance inside the medium is expected to follow an exponential decay given by the Beer-Lambert law[3]. At conditions where light attenuation is negligible - as in current experiments, where absorption is a factor of 100 smaller than scattering [4] - the attenuation coefficient is solely due to scattering. Thus, from the plot of the collected light versus the medium thickness, it is possible to determine the scattering coefficient μ_s with a single exponential fitting (Figure S4). Importantly, to ensure a single-scattering regime, the attenuation factor is measured within the region where the light intensity is attenuated from 1 to 0.6, where we can ensure scattered photons underwent only one scattering event. Once μ_s is determined, the optical thickness (τ) of the medium is calculated as $\tau = \mu_s d$, where d corresponds to the medium thickness the light traverses.

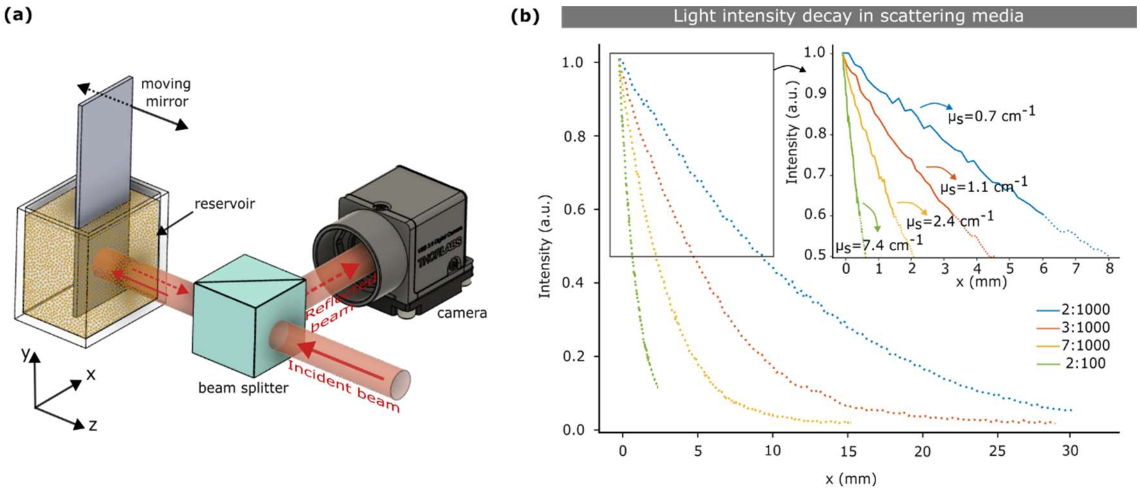

Figure S4. **Experimental measurement of the scattering coefficient of the water/milk mixtures.** (a) Scheme of the setup used to perform the measurement. The mirror was moved using a motorized translation stage (not shown). (b) Plot of the light intensity versus the thickness of water/milk mixtures. The inset also shows the computed scattering coefficient of each mixture. The mixtures correspond to milk/water dilutions of (--) 2:1000, (--) 3:1000, (--) 7:1000, and (--) 2:100.

S5- Beam propagation measurement

To experimentally visualize the propagation of light through the ultrasound-modulated medium, we captured a series of images at various locations inside the medium using an external microscope working in reflection mode, as shown in Figure S5. In more detail, we placed a moving mirror inside the modulated medium, where the light was reflected. The focal plane of the external microscope was positioned at the output of the medium, which coincided with its input in this case. Thus, by moving the mirror a certain distance (x), the external microscope effectively scanned the beam propagation inside the modulated medium. It should be noted that when the mirror moves by a distance x, the effective change in position observed by the reflective microscope is 2x, considering that light has to propagate both in and out.

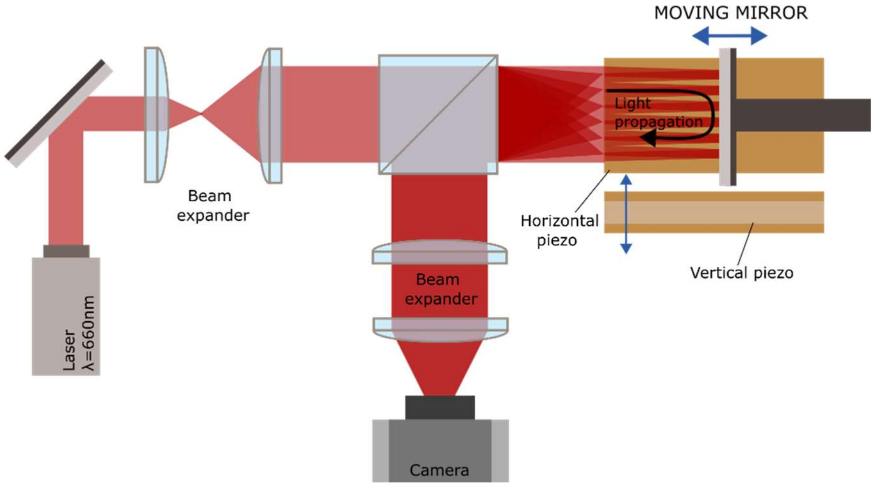

Figure S5. **Schematic of the set-up used to measure the beam propagation.** The 660 nm diode laser is directed toward the perpendicularly placed piezoelectric plates. The beam is reflected by the moving mirror and then it is captured at effectively different positions by a reflective external microscope.

S6- Beam guiding at different pressure wave amplitudes

Besides the results shown in the main paper, we also characterized the propagation of light inside the ultrasound-modulated media at additional pressure wave amplitudes, corresponding to refractive index changes ranging from $\Delta n=1\cdot10^{-5}$ to $\Delta n=10\cdot10^{-5}$, as shown in Figure S6 (see section S2 for the relationship between pressure and refractive index). In this case, the input voltage applied to the piezoelectric transducer was systematically increased, from 100 mV to 1V before amplification (Figure S1). Notably, increasing the pressure wave amplitude leads to a progressive enhancement of light focusing. Thus, light is focused after propagating shorter distances, from 20 mm to 12 mm. Given that the extent of the modulated medium is 20 mm, $\Delta n>5\cdot10^{-5}$ causes the beam to focus inside the modulated media. When this occurs, light reaching the output of the medium is slightly defocused, causing a drop in light intensity. Besides a shift in the position of the light focuses, increasing the pressure wave amplitude also leads to higher spatial confinement of the multiple-beamlets, with a spot size progressing decreasing from 160 µm to 35 µm. These results are in excellent agreement with simulations using the Beam Propagation Method (BPM).

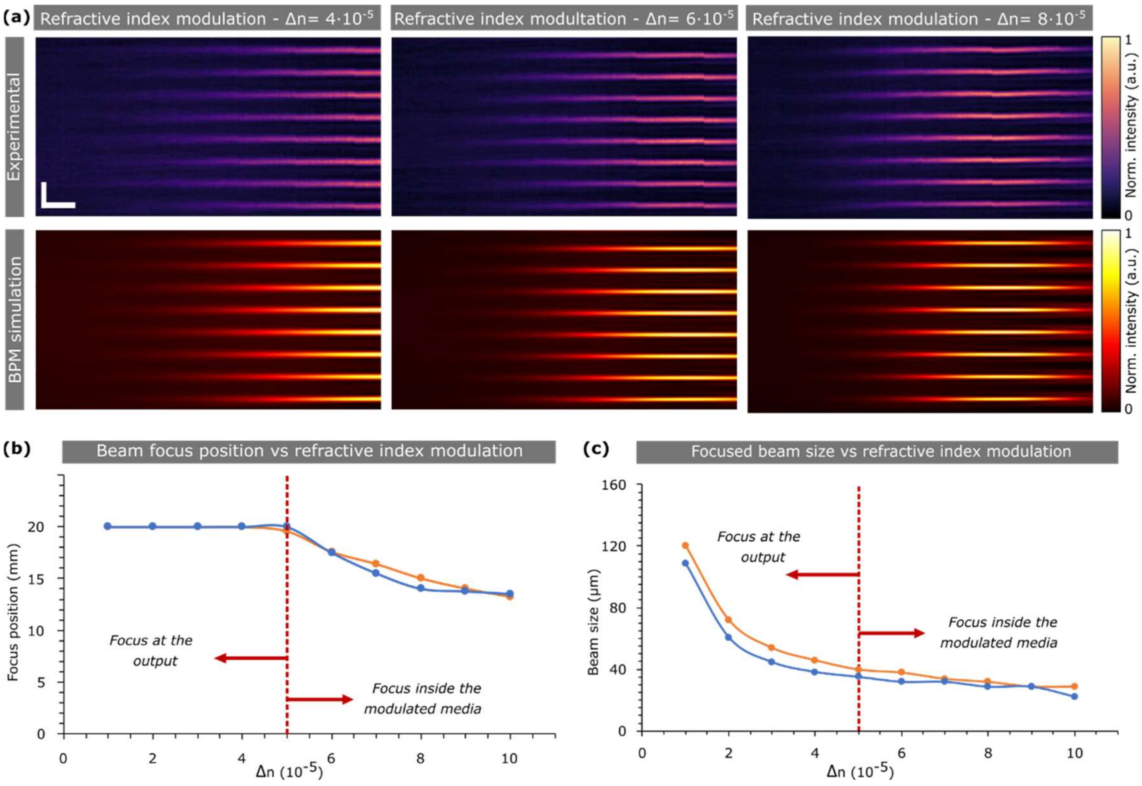

Figure S6. **Light guiding in ultrasound-modulated media at different pressure amplitudes**. (a) Experimental (upper) and simulation (lower) beam propagation images at the following working conditions: $\Delta n=4\cdot10^{-5}$, $\Delta n=6\cdot10^{-5}$, $\Delta n=10\cdot10^{-5}$. Scale bars are 200 µm (vertical) and 2 mm (horizontal). Plot of the focused beam position (b) and focused beam size (c) regarding the amplitude of the refractive index modulation. Orange data refers to simulation measurements, blue data corresponds to experimental measurements.

S7- Light intensity enhancement with ultrasound waveguiding

In addition to the results reported in the main paper, we provide further Monte Carlo simulation results on light propagation in scattering media, with and without parallelized ultrasound waveguiding. These details complement the demonstration of light enhancement and the resulting increase in penetration depth. Figures S7.1 and S7.2 show slices of the light propagation in scattering media – with optical thicknesses of 4 and 8 respectively - without (top) and with (down) ultrasound guiding. As clearly observed, ultrasound guiding enhances light intensity during propagation, leading to an increase in penetration depth, as reported in the intensity plots in Figures S7.1 and S7.2.

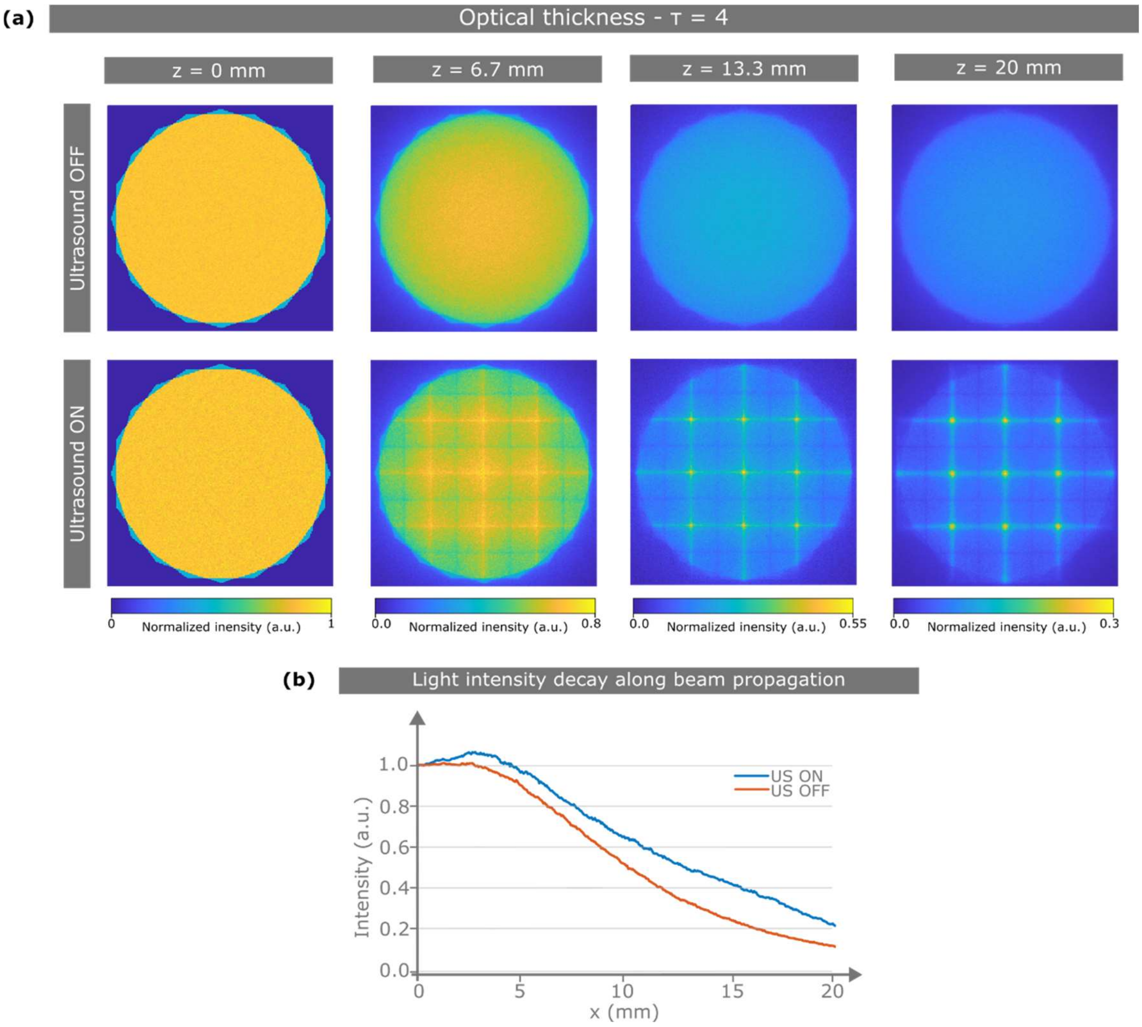

***Figure S7.1*. Monte Carlo simulation in a scattering medium of τ = 4. (a)** Slices of the light propagation along a 20 mm scattering medium without (top) and with (down) ultrasound waveguiding. **(b)** Plot of the light intensity at different positions along the light propagation in the scattering samples with (blue data) and without (orange data) ultrasound waves.

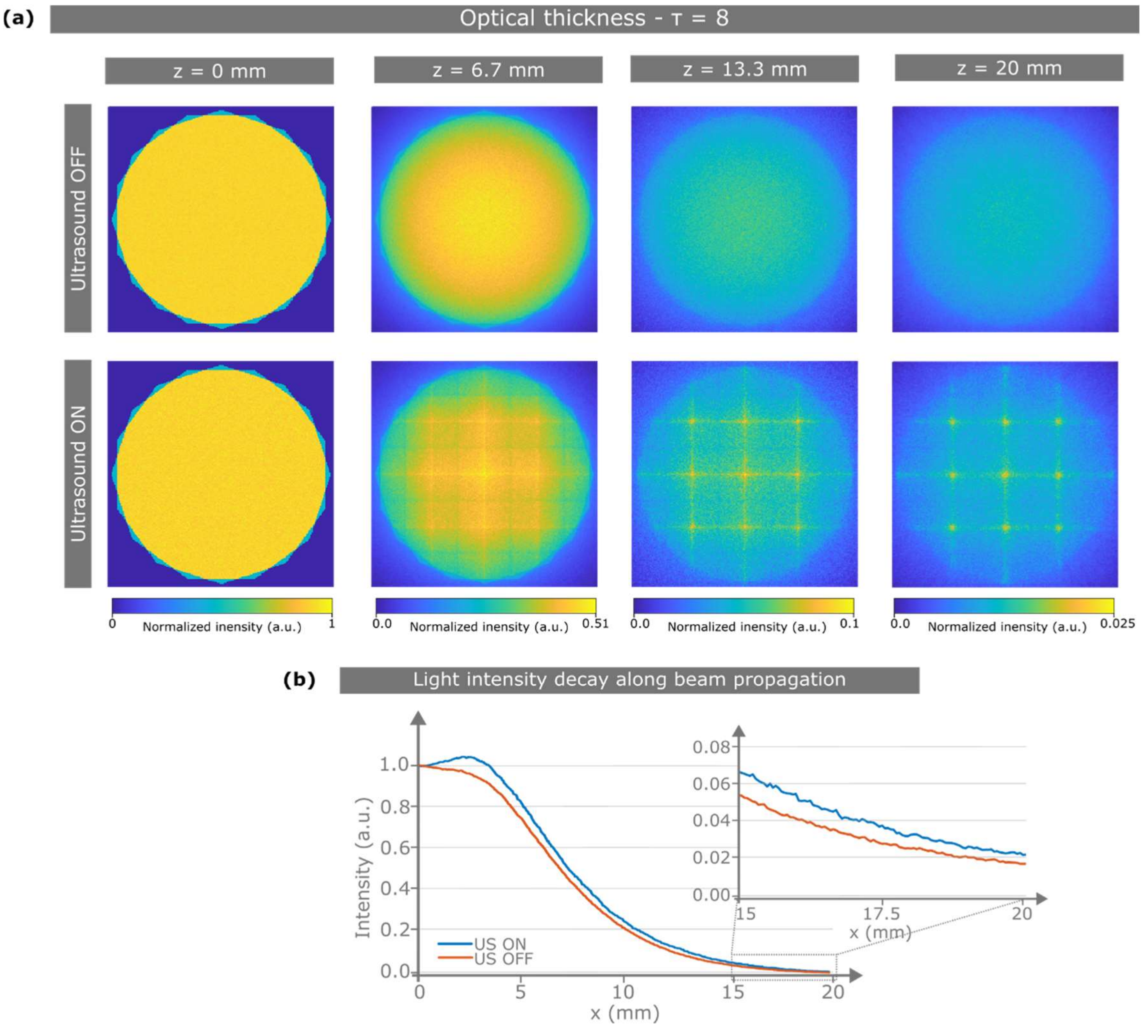

Figure S7.2. **Monte Carlo simulation in scattering medium of τ = 8. (a)** Slices of the light propagation along a 20 mm scattering medium without (top) and with (down) ultrasound waveguiding. **(b)** Plot of the light intensity at different positions along the light propagation in the scattering samples with (blue data) and without (orange data) ultrasound waves.

S8- Beam Propagation Method (BPM) simulations in scattering media

To further validate the ultrasound-waveguiding method in scattering media, we performed additional simulations using the Beam Propagation Method (BPM) [5] implemented in Python: Light Pipes for Python [6]. In this case, we used a refractive index modulation amplitude of $n_A=6\cdot10^{-5}$, and a random distribution of scattering elements with a refractive index of 1.57 and a volume of 1 μm^3 around the propagation media. With these parameters, and based on Mie scattering, the same scattering properties as the milk solution experimentally used can be reproduced. Thus, we simulated the light propagation for samples with an optical thickness of 0, 4, 8, 10.5, and 12.5 – see Figure S8. These simulations confirm the overall trend of light intensity enhancement with the use of ultrasound waves, in agreement with both Monte Carlo simulations and experiments, thereby supporting the ultrasound-waveguiding phenomenon proposed in this work.

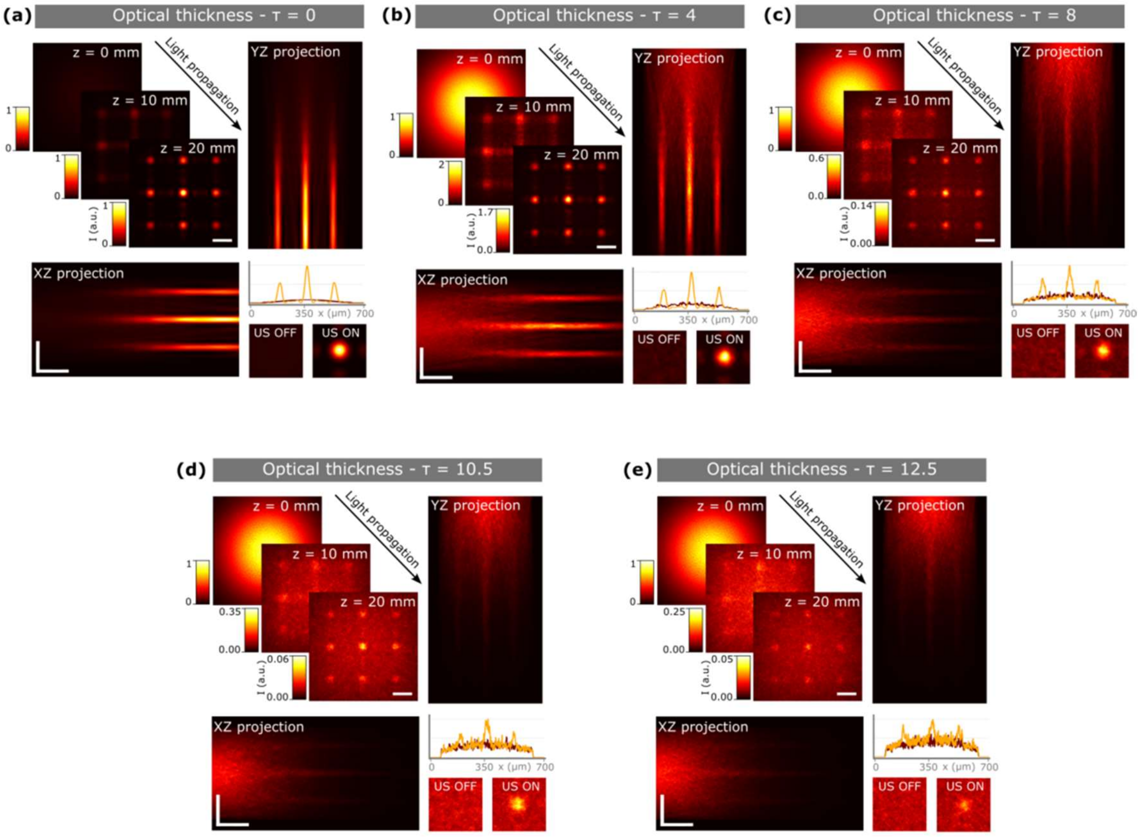

***Figure S8*. Beam Propagation Method (BPM) simulations in scattering media with ultrasound modulation.** Intensity images corresponding to BPM simulations of the beam propagation inside the ultrasound-modulated scattering media at optical thicknesses of 0 (a), 4 (b), 8 (c), 10.5 (d), and 12.5 (e). For each case, the beam at propagation distances of z = 0 mm, z = 10 mm, and z = 20 mm is shown. Scale bars are 100 µm. YZ and XZ propagation projections with the normalized output beam intensity profile (orange data corresponds to ultrasound ON, purple data corresponds to ultrasound OFF). The insets show zoomed-in intensity images at the intersecting point between two ultrasound maxima. Horizontal scale bars are 3 mm and vertical scale bars are 200 µm.

REFERENCES

1. DiComo, G. P. & Caron, J. N. Frequency response of optical beam deflection by ultrasound in water. *Appl. Optics* ***53*** 7677–7683 (2014).

2. IAPWS R9-97, Release on the Refractive Index of Ordinary Water Substance as a Function of Wavelength, Temperature and Pressure (1997). https://iapws.org/relguide/rindex.pdf (accessed 2024/11/04)

3. Bhatt, M., Ayyalasomayajula, K. R. & Yalavarthy, P. K. Generalized Beer–Lambert model for near-infrared light propagation in thick biological tissues. *J Biomed Opt* **21**, 076012 (2016).

4. Aernouts, B. *et al.* Visible and near-infrared bulk optical properties of raw milk. *J Dairy Sci* **98**, 6727–6738 (2015).

5. Glaser, A. K., Chen, Y. & Liu, J. T. C. Fractal propagation method enables realistic optical microscopy simulations in biological tissues. *Optica* **3**, 861 (2016).

6. Vdovin, G., Van Goor, F. LightPipes for Python. https://opticspy.github.io/lightpipes/index.html (accessed 2024/11/04).
